# Hotspots of Biased Nucleotide Substitutions in Human Genes

**DOI:** 10.1371/journal.pbio.1000026

**Published:** 2009-01-27

**Authors:** Jonas Berglund, Katherine S Pollard, Matthew T Webster

**Affiliations:** 1 Department of Medical Biochemistry and Microbiology, Uppsala University, Uppsala, Sweden; 2 Gladstone Institutes, University of California, San Francisco, California, United States of America; University of Bath, United Kingdom

## Abstract

Genes that have experienced accelerated evolutionary rates on the human lineage during recent evolution are candidates for involvement in human-specific adaptations. To determine the forces that cause increased evolutionary rates in certain genes, we analyzed alignments of 10,238 human genes to their orthologues in chimpanzee and macaque. Using a likelihood ratio test, we identified protein-coding sequences with an accelerated rate of base substitutions along the human lineage. Exons evolving at a fast rate in humans have a significant tendency to contain clusters of AT-to-GC (weak-to-strong) biased substitutions. This pattern is also observed in noncoding sequence flanking rapidly evolving exons. Accelerated exons occur in regions with elevated male recombination rates and exhibit an excess of nonsynonymous substitutions relative to the genomic average. We next analyzed genes with significantly elevated ratios of nonsynonymous to synonymous rates of base substitution (*d*_N_ */d*_S_) along the human lineage, and those with an excess of amino acid replacement substitutions relative to human polymorphism. These genes also show evidence of clusters of weak-to-strong biased substitutions. These findings indicate that a recombination-associated process, such as biased gene conversion (BGC), is driving fixation of GC alleles in the human genome. This process can lead to accelerated evolution in coding sequences and excess amino acid replacement substitutions, thereby generating significant results for tests of positive selection.

## Author Summary

Regions of the human genome that appear to evolve rapidly may have been under strong positive selection and could contain the genetic changes responsible for the uniqueness of our species. However, neutral (nonadaptive) evolutionary processes can give rise to signals that can be mistaken as signs of selection. In this article, we identify coding sequences that have undergone accelerated rates of change in humans, affecting the divergence of the proteins they encode. By analyzing patterns of molecular evolution in these genes and their distribution in the genome, we show that many protein-coding changes in the fastest-changing genes are not a result of selection operating on the genes, but instead result from biased fixation of AT-to-GC mutations. Our findings are consistent with a model of recombination-driven biased gene conversion. This leads to the provocative hypothesis that many of the genetic changes leading to human-specific characters may have been prompted by fixation of deleterious mutations.

## Abstract 3

Natural selection is commonly believed to be the main engine of functional genetic change, but a separate neutral evolutionary process linked to recombination may have contributed significantly to the divergence of human proteins.

## Introduction

Whole-genome comparisons have revealed hundreds of noncoding elements that are extremely conserved across mammals but show evidence for accelerated evolution along the human lineage [1–4]. One possible explanation for these human-accelerated regions (HARs) is the action of positive selection in the human lineage. However, HARs tend to have biased patterns of nucleotide substitution, dominated by AT → GC changes—referred to here as “weak-to-strong” (W→S) as they result in a replacement of a “weak” A:T bond with a “strong” G:C bond. This pattern is strongly discordant with the genomic average, where S→W substitutions predominate. Positive selection is not expected to generate such biased patterns of base substitution. Interestingly, HARs also have a propensity to occur in regions with high recombination rates. These biased substitution patterns could potentially be explained by variation in the pattern of mutation, localized selection for increased GC content, or by biased gene conversion (BGC), which is a recombination-associated molecular drive that favors fixation of W→S mutations [5,6] and has population dynamics similar to natural selection [7].

There is now strong evidence for an association between recombination and patterns of nucleotide substitutions in the human genome, suggesting that an excess of W→S base substitutions occur in regions of high recombination [8]. The evidence can be summarized as follows: first, patterns of substitution in human–primate genomic alignments correlate with human recombination rates [8–10]. Second, parts of mammalian and avian genomes subject to very high recombination rates, such as duplicated gene families [11–13] and the X-linked pseudoautosomal region [14], are both extremely GC-rich and have GC-biased substitution patterns. Third, GC content correlates with recombination in a wide range of eukaryotes [15–17]. Fourth, experiments on primate cell lines and yeast indicate a bias in repair mechanisms, which leads to mismatches being preferentially repaired to GC bases [15,18,19]. Fifth, GC-biased clustered substitutions have been observed close to human recombination hotspots and near telomeres [20], and these regions also tend to be more GC-rich [21].

Several studies have also reported a correlation between sequence divergence and recombination rate [22–24]. It is therefore possible that recombination could directly influence patterns of substitution, although it is unknown whether mutations generated by recombination are W→S biased. The fact that the proportion of W→S mutations leading to human single-nucleotide polymorphisms (SNPs) is discordant with the proportion leading to nucleotide substitutions on the human lineage [25] strongly suggests a bias in fixation rather than mutation processes. Further support comes from observations that W→S and S→W changes segregate at different frequencies on average in human populations, particularly in regions with elevated recombination rates [6,25,26] (but see [27]).

The distribution of recombination events is highly variable along vertebrate chromosomes. Recombination is mainly restricted to short (<1 kb) hotspots [28], which are extremely short-lived over evolutionary time [29]. Clusters of W→S biased substitutions have been observed on a similar scale, and it is proposed that these are the result of biased fixation of GC alleles in recombination hotspots [20]. Recombination hotspots therefore could be responsible for localized lineage-specific bursts of W→S biased substitutions, which could contribute to human-accelerated evolution in conserved noncoding elements.

Biased substitution patterns could also potentially result from selection on GC content. Mammalian genomes exhibit variation in GC content on the scale of hundreds of kilobases, commonly referred to as the isochore structure [30,31]. A potential explanation for this variation is that some regions experience selection in favor of increased GC content due to increased thermal stability [30]. In addition to this, experimental evidence indicates that GC-rich genes may be expressed with greater efficiency than GC-poor genes [32], which could lead to selection in favor of increased GC content in expressed sequences. Selection is more efficient on regions of high recombination due to a reduction in Hill-Robertson interference [33], which could lead to increased fixation of W→S mutations in these regions due to selection.

Some protein coding sequences also show patterns of evolution that are consistent with high levels of recombination-associated fixation of W→S mutations. For example, the *Fxy* gene is found on the X-specific portion of the X chromosome in human, rat, and short-tailed mouse (Mus spretus). However, in the house mouse (M. musculus), this gene has been translocated so that only its 5′ portion now resides in the X-specific region, whereas its 3′ portion overlaps the pseudoautosomal region (PAR), which is subject to very high levels of recombination [14]. This translocation resulted in a massive increase in substitution rate in the PAR portion of the gene, including substitutions that cause amino acid replacements. Since the common ancestor of M. spretus and *M. musculus*, the M. spretus lineage has accumulated one replacement substitution in the 3′ portion and one replacement substitution in the 5′ portion. In contrast, the M. musculus lineage has accumulated no substitutions in the 5′ portion, but 28 replacement substitutions in the PAR-overlapping 3′ portion [5]. Furthermore, all 28 substitutions are W→S.

Substitution patterns in the HARs and the *Fxy* gene may indicate that recombination-associated biased fixation of W→S mutations can compete with purifying selection, leading to the accumulation of weakly deleterious variants [5]. It is possible that this effect could affect common tests for positive selection in coding sequences, although the predicted impact of W→S fixation bias on these tests has not been demonstrated. For example, a gene under the influence of W→S fixation bias on a particular lineage could potentially acquire an increased ratio of nonsynonymous to synonymous rates of base substitution (*d*_N_ */d*_S_). This could lead to a significant acceleration in *d*_N_ */d*_S_, which is commonly assumed to indicate positive selection [34]. Similarly, fixation of weakly deleterious variants could potentially lead to an excess of amino acid replacement substitutions compared with polymorphism. This could generate significant results for the McDonald-Kreitman [35] test of neutrality, which could also lead to false inference of positive selection on protein sequence.

We examined the possibility that biased fixation of W→S mutations could affect the evolution of human protein-coding regions across the genome by analyzing patterns of evolution in a genome-wide set of human-chimpanzee-macaque 1:1:1 orthologous genes [36]. We first identified individual protein-coding exons with evidence for accelerated rates of nucleotide substitution in the human lineage using a likelihood ratio test (LRT). We then characterized patterns of nucleotide substitution in these loci. We also tested whether fast-evolving genes are associated with recombination hotspots or regions of elevated recombination. Our findings are consistent with the hypothesis that a recombination-associated process has generated an increased rate of nucleotide substitutions on the human lineage within particular genes since the split with chimpanzee. Interestingly, these genes also have increased numbers of amino acid replacement substitutions. This observation motivated us to theoretically and empirically examine the relationship between W→S fixation bias and rates of nonsynonymous base substitution. Our results suggest that W→S fixation bias can generate significant results for tests designed to detect directional selection, including LRTs for accelerated *d*_N_ */d*_S_ [34] and McDonald-Kreitman tests [35], potentially leading to false inference of positive selection.

## Results

We analyzed a dataset of 10,238 genes for evidence of accelerated evolutionary rates. We first divided the genes into 84,784 exons and used a LRT to identify individual exons with evidence for an increased rate of nucleotide substitution on the human lineage, considering both synonymous and nonsynonymous substitutions. Using this approach, we were able to identify the effects of local increases in substitution rate over a scale of ∼1 kb, which are likely to affect only single exons and nearby flanking sequence. Significance was evaluated by simulating 10,000 datasets based on the null model of no acceleration, and correcting for multiple testing using the method of Benjamini and Hochberg [37]. The entire dataset is presented in Table S1.

### Accelerated Exons Have Biased Patterns of Base Substitution

In total, 83 exons (in 82 genes) show evidence for acceleration in the human lineage with an expected false discovery rate (FDR) less than 5%. Henceforth, we refer to these 83 human-accelerated coding sequences as “accelerated exons.” The mean length of the accelerated exons is 516.6 bp, which is higher than the mean length of exons in the entire dataset (167.6 bp). On average, the accelerated exons contain 7.73 substitutions on the human lineage, compared to a mean of 0.43 substitutions in the entire dataset. This suggests that the majority of exons are too short for an acceleration in evolutionary rate to be detectable by this method, given the evolutionary distance between human and chimpanzee.

Genes containing the accelerated exons have a mean of 13.3 human substitutions, compared with an average of 3.58 in all genes. Furthermore, there is a tendency for substitutions in these genes to be clustered: 11.0% of genes containing accelerated exons have a significantly nonuniform clustering of substitutions into the most diverged exon (*p* < 0.05), compared to less than 1.1% of all genes (see Methods).

**Figure 1:**
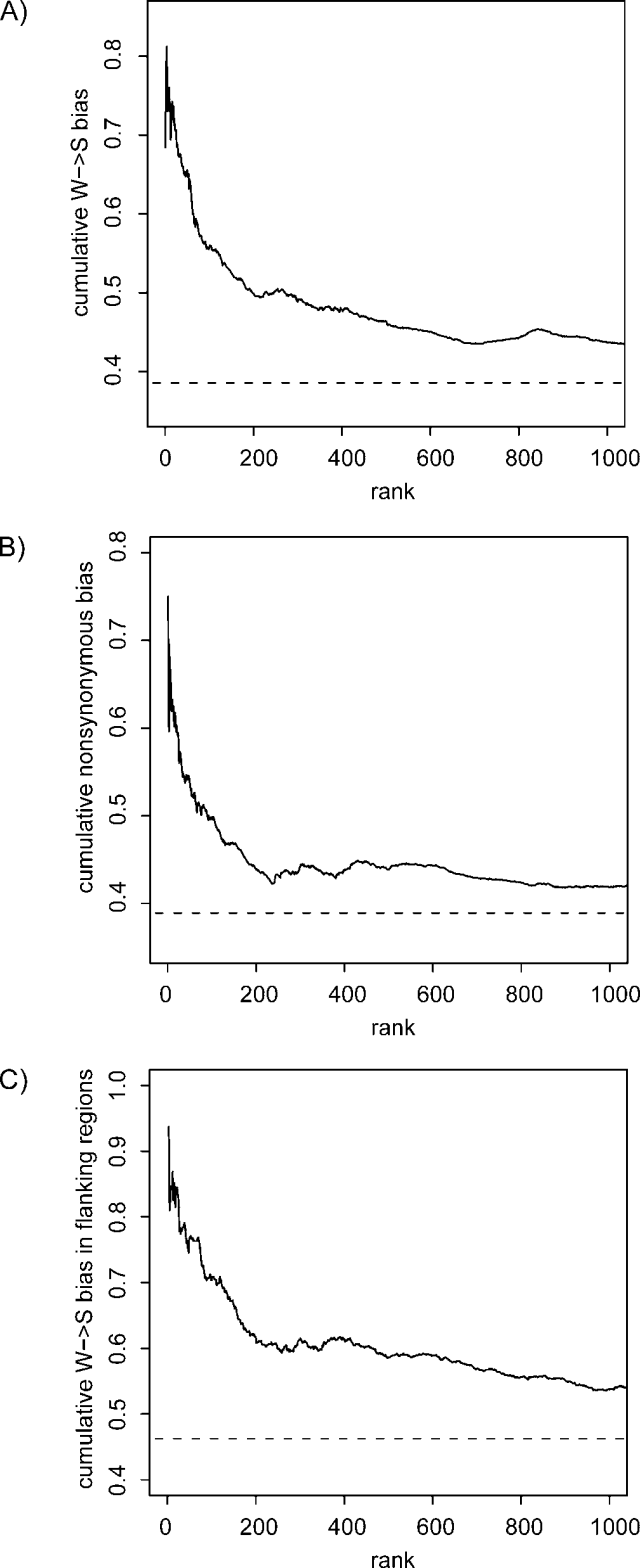
Cumulative Bias in Different Classes of Nucleotide Substitutions in the Exons with the Highest Degree of Acceleration on the Human Lineage (A) The proportion of W→S substitutions compared to W→S and S→W substitutions on the human lineage. (B) The proportion of nonsynonymous substitutions compared to all substitutions in each gene on the human lineage. (C) The proportion of W→S substitutions compared to W→S and S→W substitutions on the human lineage in the flanking noncoding regions. Dashed lines represent averages for the entire dataset.

**Figure 2:**
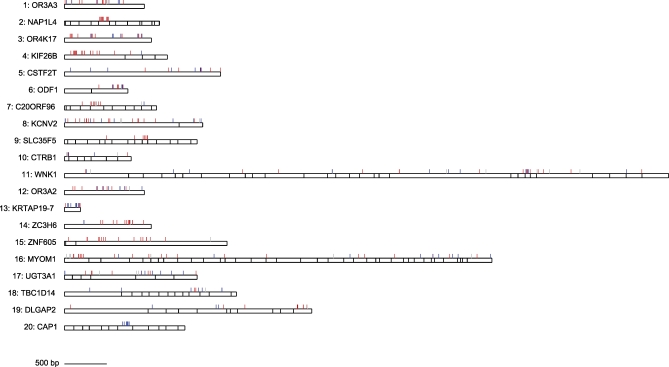
Genes Containing the Most Accelerated Exons Exon boundaries are marked with black lines. S→W substitutions on the human lineage are marked with blue lines, W→S substitutions on the human lineage are marked with red lines, and all other substitutions on the human lineage are marked with grey lines.

We estimated the pattern of substitution on the human and chimpanzee lineages by comparing the extant sequences with the maximum likelihood (ML) reconstructed ancestor using a codon model of substitution where the *d*_N_ */d*_S_ on the human branch was able to vary from the rest of the tree. The 83 accelerated exons demonstrate a bias for weak to strong (W→S) substitutions, with 326 W→S and 248 S→W base substitutions (W→S bias = 0.57; see Methods). This substitution pattern is significantly incongruent with the genome as a whole, where W→S bias = 0.39 (Fisher's exact test (FET) *p* < 2.2 × 10^−16^; bootstrap *p* < 0.001). This bias strongly affects the most accelerated exons and drops to close to the genomic average for exons with less evidence of acceleration (Figure 1A). Strikingly, the top 20 accelerated exons (Table 1) have 154 W→S compared to only 62 S→W substitutions (W→S bias = 0.71, FET *p* < 2.2 × 10^−16^; bootstrap *p* < 0.001). The positions of each nucleotide substitution on the human lineage in the genes containing the top 20 accelerated exons are shown in Figure 2. The excess of W→S substitutions can be clearly seen, and there is an obvious tendency for clusters of W→S substitutions to occur in single exons in particular genes.

**Table 1:**
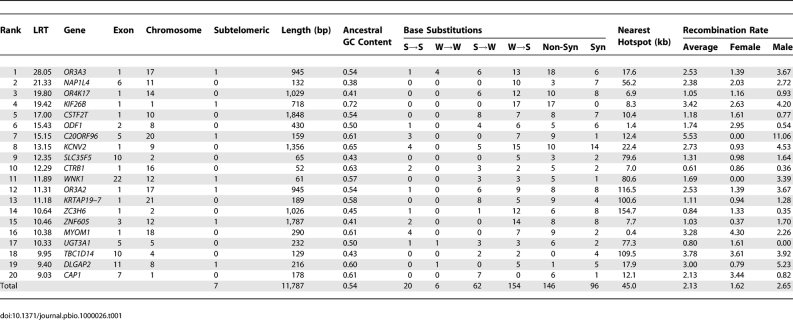
Patterns of Nucleotide Substitution in the Top 20 Accelerated Exons

We used the ML reconstructed human-chimpanzee ancestral sequences to infer the ancestral GC content of each exon. The accelerated exons have an average ancestral GC content of 0.53, whereas the top 20 accelerated exons have an average GC content of 0.54. In comparison, average ancestral GC content in all of the coding sequences in the dataset is 0.50. The elevated GC content of accelerated exons is observed at all three codon positions (unpublished data). Differences in GC content therefore cannot explain the differences in base substitution patterns between accelerated and nonaccelerated genes. If base substitution probabilities were constant across genomic regions, genes with higher GC content would be expected to have lower W→S substitution rates, when the opposite is actually observed. Our test for differences in the substitution patterns is therefore conservative.

The most accelerated exons also have a bias toward a greater number of nonsynonymous substitutions compared with the genomic average. The proportion of nonsynonymous substitutions in the top 20 accelerated exons is 0.60 (146 nonsynonymous versus 96 synonymous), which is significantly higher than the proportion of 0.38 observed in the entire dataset (FET *p* < 3.0 × 10^−11^; bootstrap *p* < 0.001). This bias is most extreme for the most accelerated exons (Figure 1B). Nonsynonymous substitutions also have a tendency to exhibit a stronger W→S bias, particularly in the accelerated exons. The W→S bias of accelerated exons is 0.63 for nonsynonymous sites and 0.51 for synonymous sites (FET *p* = 0.004). In the top 20 accelerated exons, W→S bias is 0.75 for nonsynonymous sites and 0.66 for synonymous sites (FET *p* = 0.129). In the entire dataset, W→S bias is 0.41 for nonsynonymous sites and 0.37 for synonymous sites (FET *p* = 2 × 10^−9^).

### Flanking Noncoding Sequences Also Exhibit Biased Patterns of Base Substitution

To determine whether the W→S substitution bias is confined to protein-coding regions, we analyzed the pattern of base substitution in noncoding regions within 100 bp flanking both sides of each accelerated exon. Around the top 20 accelerated exons, there are 60 W→S but only 11 S→W base substitutions (W→S bias = 0.85). This is significantly different from the flanking regions surrounding all exons in the dataset (W→S bias = 0.46; FET *p* < 3.2 × 10^−11^; bootstrap *p* < 0.001). The W→S bias in flanking sequences is strongest for the most accelerated exons (Figure 1C). Average ancestral GC content in the regions flanking the top 20 accelerated exons is 0.48, which is higher than the average of 0.44 in the entire dataset. This suggests that differences in GC content could not be responsible for the differences in patterns of base substitution.

**Figure 3:**
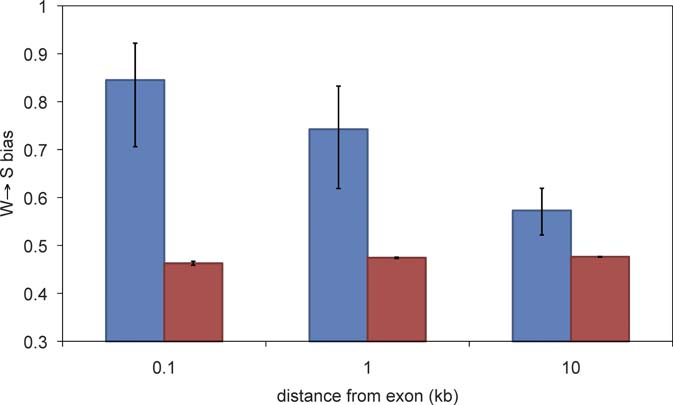
Patterns of Base Substitution in Flanking Regions Average W→S bias of base substitution patterns in noncoding regions surrounding the top 20 accelerated exons (blue bars) compared with W→S bias in noncoding regions surrounding all exons in the dataset (red bars). 95% confidence intervals were estimated by bootstrapping with 1,000 replicates.

We analyzed patterns of substitution in progressively larger windows of noncoding sequence surrounding each exon to determine the scale at which the W→S bias in substitution patterns exists (Figure 3). We found that there is a marked decrease in W→S bias with increasing distance from the accelerated exon. The W→S bias in 10 kb of noncoding sequence on both sides of the 20 most accelerated exons approaches the genomic average. These results suggest that the process generating the W→S bias in substitution patterns in accelerated exons acts on a regional level, rather than specifically on coding sequences. Furthermore, strongly W→S biased substitution patterns seem to be restricted to a scale of less than a few kilobases.

### Accelerated Exons Occur in Regions of Elevated Male Recombination

**Figure 4:**
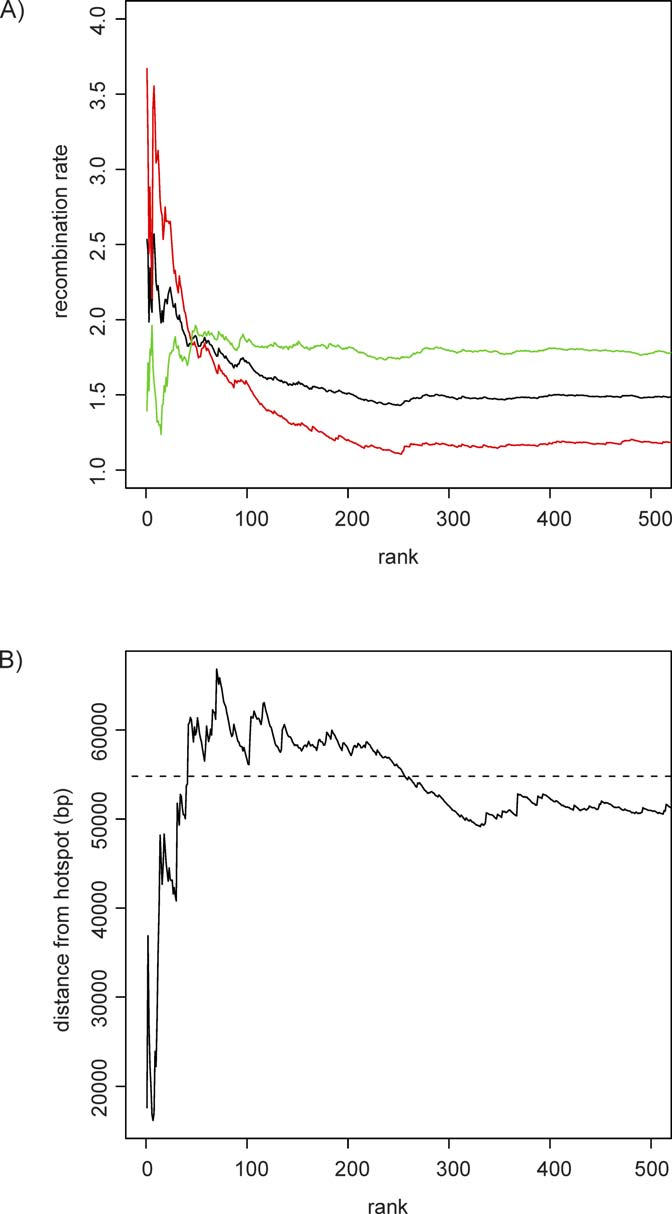
Cumulative Average Human Recombination Rate in the Regions Surrounding the Most Accelerated Exons (A) Cumulative average male (red), female (green), and sex-averaged (black) recombination rates. (B) Cumulative average distance to the nearest recombination hotspot. The dashed line represents the average for the entire dataset.

We investigated the recombination rates of the regions where accelerated exons reside. We find that the most accelerated exons tend to be found in regions with elevated male recombination rates. In the top 20 accelerated exons, the average male recombination rate is 2.65, which is significantly higher than the average of all exons in the dataset (0.92; bootstrap *p* < 0.001). By contrast, female recombination rate in the top 20 accelerated exons is 1.62, which is not significantly higher than the genomic average of 1.69 (bootstrap *p* = 0.45). Male recombination rate is therefore highly elevated in the most accelerated exons, whereas female recombination rate remains relatively constant (Figure 4A).

Accelerated exons show a slight tendency to occur near human recombination hotspots (Figure 4B). In the top 20 accelerated exons, average distance to a hotspot is 50.0 kb, compared to 54.8 kb in the entire dataset, although this difference is not significant (bootstrap *p* = 0.16). There is a highly significant tendency for accelerated exons to be found close to telomeres, where recombination rates are elevated in males [17]. Seven out of the top 20 accelerated exons are found in the last chromosome band. This proportion (0.35) is significantly higher than 0.075 observed in the entire dataset (bootstrap *p* < 0.001).

### Exons That Are Highly Diverged Compared to the Rest of the Gene Have Biased Substitution Patterns

We identified genes where one exon had greater divergence on the human lineage than the rest of the coding sequence, termed “relative divergence” (see Methods). Relative divergence was not calculated for exons with less than four substitutions to avoid bias from very short sequences. The 20 genes showing the highest relative divergence have most diverged exons with a significant excess of W→S substitutions compared with the entire dataset (FET *p* = 0.00013; Table 2). The spatial distribution of substitutions in the genes containing the top 20 most relatively diverged exons is presented in Figure S1. This pattern is also observed in the flanking regions of highly relatively diverged exons compared with the entire dataset (FET *p* = 2.3 × 10^−9^). Thus, when genes contain clusters of nucleotide substitutions in single exons, these substitutions tend to exhibit a W→S biased pattern. The observation that the patterns extend into flanking introns and intergenic sequence suggests that these patterns do not result from selection on protein coding sequence and that a regional bias in mutation or fixation of mutations is a more likely explanation.

**Table 2:**
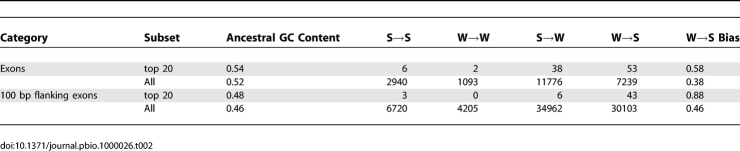
Patterns of Nucleotide Substitution in Exons with High Divergence Compared to the Remaining Exons of the Same Gene

### Patterns of Nucleotide Divergence and Polymorphism Are Discordant in Accelerated Exons

To distinguish between biases in patterns of mutation and fixation, we compared W→S bias in nucleotide substitutions with human SNPs from the HapMap project. Patterns of substitutions in accelerated exons are significantly more W→S biased than human SNPs (Table 3). These differences are highly significant for the top 20 accelerated exons (FET *p* = 0.0015) and for all 83 accelerated exons (FET *p* = 0.00063). These results suggest that either the W→S bias substitution patterns in these exons result from a mutation bias that is no longer active in the human population, or that the patterns result from a bias towards fixation of W→S mutations.

**Table 3:**
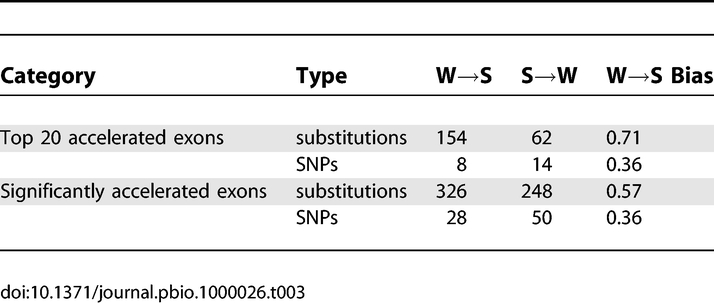
Patterns of Nucleotide Substitutions Compared with SNPs in Human-Accelerated Exons

### Exons with Elevated *d*_N_/*d*_S_ in Humans Have Biased Substitution Patterns

We performed an additional LRT to identify individual exons with significantly elevated *d*_N_ */d*_S_ ratios on the human lineage. On average, we inferred 0.17 nonsynonymous and 0.26 synonymous substitutions per exon since the human-chimpanzee split, which suggests that estimates of *d*_N_ */d*_S_ are unreliable for most exons. We therefore restricted our analysis to exons with more than four substitutions inferred on the human lineage. Only 887 exons out of the entire dataset (*n* = 84,784) passed this criterion (1.0%). As shown in Table 4, exons with evidence for accelerated *d*_N_ */d*_S_ in humans tend to have more W→S biased patterns of nucleotide substitution. At the *p* < 0.01 (**) level, this is significant by FET (*p* = 0.015) but not bootstrap (*p* = 0.104). At the *p* < 0.05 (*) level, neither of the tests are significant (FET *p* = 0.104; bootstrap *p* = 0.130). Exons with accelerated *d*_N_ */d*_S_ on the human lineage therefore appear to be associated with W→S biased patterns of nucleotide substitution, although in the majority of exons, not enough substitutions have occurred to perform this test.

**Table 4:**
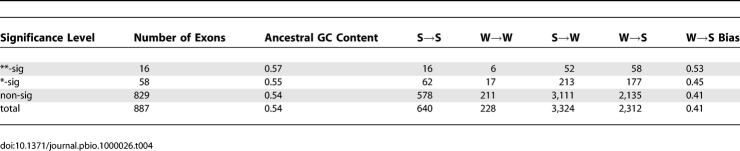
Patterns of Nucleotide Substitution in Exons with Evidence for Elevated *d*_N_ */d*_S_ on the Human Lineage

### Patterns of Base Substitution in Accelerated Exons Are Also Biased in Chimpanzee

The 83 accelerated exons have a significantly higher number of substitutions than average on the chimpanzee lineage. There is a base substitution in 0.0043 of sites on the chimpanzee lineage in the top 20 accelerated exons, compared to 0.0011 in all exons on the chimpanzee lineage (FET *p* < 2.2 × 10^−16^; bootstrap *p* = 0.001). This is an interesting observation, given that the LRT is designed to identify acceleration specifically on the human branch.

Accelerated exons show similar, although less pronounced, patterns of W→S biased substitutions in the chimpanzee lineage. The top 20 accelerated exons are inferred to have a W→S bias of 0.50, compared with 0.39 averaged across all exons. However, these values are not significantly different by FET (*p* = 0.053) or bootstrap (*p* = 0.31). The top 20 accelerated exons also have a greater-than-average proportion of nonsynonymous substitutions in the chimpanzee lineage, with 54 nonsynonymous and 30 synonymous substitutions, a bias of 0.64 toward nonsynonymous substitutions, compared with 0.42 in the entire dataset (FET *p* < 5.35 × 10^−5^; bootstrap *p* = 0.002). There is also a similar W→S bias in the noncoding regions flanking accelerated exons in the chimpanzee genome, with 18 W→S and 11 S→W substitutions (W→S bias = 0.62) compared with a W→S bias of 0.46 in all of the flanking sequences (FET *p* = 0.093; bootstrap *p* = 0.146). These results are consistent with previous studies suggesting that regional patterns of base substitution are correlated between human and chimpanzee [10,20,38].

### Genes with Elevated *d*_N_/*d*_S_ in Humans Have Biased Substitution Patterns

We next examined the relationship between rates of protein evolution and W→S substitution bias on the whole-gene level. We identified genes with significant evidence for accelerated nonsynonymous substitution rates on the human lineage using branch models of codon substitution and a LRT. We refer to these as “genes with accelerated *d*_N_ */d*_S_”. Based on significance of rejection of a model with single *d*_N_ */d*_S_ ratio (codeml model 0) by a model where the human lineage had a separate *d*_N_ */d*_S_ (codeml model 2; see Methods), we defined three different levels of significance: *p* < 0.001 (***; 20 genes), *p* < 0.01 (**; 112 genes) and *p* < 0.05 (*; 485 genes). Table 5 shows the pattern of nucleotide substitution in genes at each level of significance. The distribution of these substitutions in the top 20 genes is presented in Figure S2. LRT statistics for all genes in our dataset are presented in Table S2.

**Table 5:**
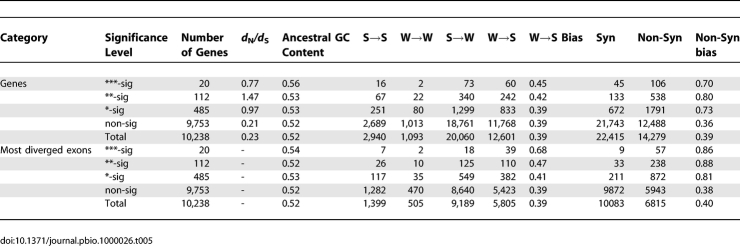
Patterns of Nucleotide Substitution in Genes with Human-Accelerated *d*_N_ */d*_S_

Comparison of the substitution patterns in genes with accelerated *d*_N_ */d*_S_ to the entire dataset reveals a trend towards W→S biased substitution patterns, although this is not significant. However, when we restrict the analysis to the exon in each gene with the largest number of human substitutions per base, the substitution pattern in *d*_N_ */d*_S_ category *** exhibits a much higher W→S bias (0.68) than the average W→S bias for most diverged exons (0.39) in the entire dataset. This difference is highly statistically significant (FET *p* = 7.1 × 10^−6^; bootstrap *p* = 0.0065). The W→S bias in the most diverged exons of genes in *d*_N_ */d*_S_ category ** is less extreme (0.47), but still significantly different from the entire dataset (FET *p* = 0.012; bootstrap *p* = 0.044). In *d*_N_ */d*_S_ category *, the average W→S bias is lower (0.41), and not significantly different from the entire dataset. In addition to being the region with the most W→S biased substitution pattern, the most diverged exon in each gene also tends to have a larger proportion of nonsynonymous substitutions (Table 5).

We noticed that the gene *KIF26B*, kinesin family member 26B, contributes disproportionately to the number of substitutions in the genes in *d*_N_ */d*_S_ category ***. *KIF26B* is located in the last band of the q arm of human chromosome 1. Its most diverged exon (exon 1) contains 17 substitutions, which are all W→S. The most diverged exons in the remaining 19 genes in *d*_N_ */d*_S_ category *** contain an average of just 2.6 substitutions. These exons have an average W→S bias of 0.55, compared with 0.68 when *KIF26B* is included, whereas the W→S bias among the most diverged exons is 0.39. The W→S bias in these 19 exons (with *KIF26B* excluded) is still significantly higher than average (FET *p* = 0.035; bootstrap *p* = 0.019). However, the evolution of *KIF26B* is particularly striking. It is the only gene that both contains one of the top 20 accelerated exons and has highly significant acceleration in *d*_N_ */d*_S_ (***). It also has a strongly W→S biased substitution pattern.

### No Evidence for W→S Bias in Noncoding Sequences Flanking Genes with Accelerated *d*_N_/*d*_S_

We examined patterns of base substitution in 100 bp of noncoding sequence flanking each side of all exons in the genes with accelerated *d*_N_ */d*_S_ (Table 6). There is no evidence that noncoding sequence nearby genes with accelerated *d*_N_ */d*_S_ at any of the levels of significance have W→S biased substitution patterns compared with genomic averages. This is the case when we analyze all the exons in genes with accelerated *d*_N_ */d*_S_ and when we analyze only the most diverged exon in each of these genes (tested by FET and bootstrap, unpublished data).

**Table 6:**
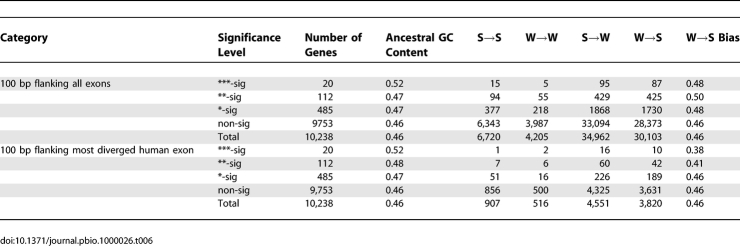
Patterns of Nucleotide Substitution in Noncoding Regions Flanking Genes with Human-Accelerated *d*_N_ */d*_S_

### Genes with Accelerated *d*_N_/*d*_S_ in Humans Are Closer to Recombination Hotspots

We examined whether genes with accelerated *d*_N_ */d*_S_ tend to be associated with regions of elevated recombination using a bootstrap test (Table 7). There is no significant tendency for accelerated genes at any of the *p*-value cutoffs to occur close to telomeres, or in regions of elevated male, female, or sex-averaged recombination. However, there is a highly significant (*p* < 10^−4^) tendency for genes in *d*_N_ */d*_S_ significance category *** (*p* < 0.001) to be closer to recombination hotspots. This tendency is also significant for genes in *d*_N_ */d*_S_ category ** (*p* = 0.032) but not for those in *d*_N_ */d*_S_ category *.

**Table 7:**
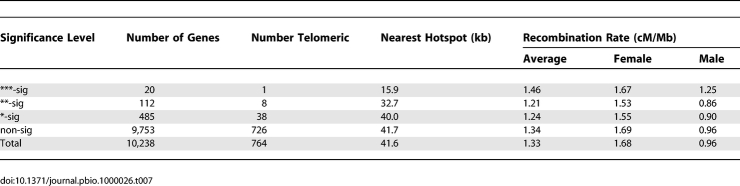
Recombination Rate in Genes with Human-Accelerated *d*_N_ */d*_S_

### McDonald-Kreitman Tests for Selection

We examined patterns of nucleotide substitution in genes with evidence for an excess of amino acid replacement substitutions on the human lineage compared to human polymorphisms. These genes were identified using a modified version of the McDonald-Kreitman (MK) test [35] presented by Bustamante et al. [39]. A total of 3,878 genes in this dataset overlapped with the human-chimpanzee-macaque orthologues. Out of these, 20 show evidence for an excess of replacement amino substitutions at the *p* < 0.01 (**) level and 124 are significant at the *p* < 0.05 (*) level (Table 8). The proportion of W→S nucleotide substitutions is clearly elevated in these genes, and this is most pronounced in the most diverged exon of each gene. For the genes with the strongest excess of amino-acid substitutions (**), this increase is not significant for substitutions across the entire gene (FET *p* = 0.055; bootstrap *p* = 0.11), but is highly significant for substitutions in the most diverged exon (FET *p* = 0.0012; bootstrap *p* = 0.008). For all genes with evidence for an excess of amino-acid substitutions (*), the W→S bias is significant for substitutions across the entire gene (FET *p* = 0.025; bootstrap *p* = 0.0446), but not for substitutions in the most diverged exon (FET *p* = 0.055; bootstrap *p* = 0.07). MK tests based on HapMap SNP data (http://www.hapmap.org/) for all genes in our dataset show similar patterns (unpublished data) but are subject to ascertainment bias and do not accurately reflect the true underlying SNP density. In summary, there is a clear association between excess amino acid replacement substitutions and W→S biased substitution patterns.

**Table 8:**
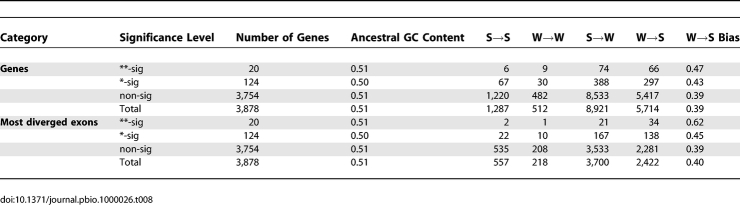
Patterns of Nucleotide Substitution in Genes with Evidence for Positive Selection Based on MK Tests

Table 9 shows the recombination rates of genes identified by the MK test. The genes with the strongest excess of nonsynonymous substitutions (**) are situated significantly closer to recombination hotspots (bootstrap *p* = 0.026) than the rest of the genes. For all significant genes, the mean distance to a hotspot is higher than average, although this is not significant (*p* = 0.765). There are no significant differences between the average or sex-specific recombination rates in genes with significant MK test values.

**Table 9:**
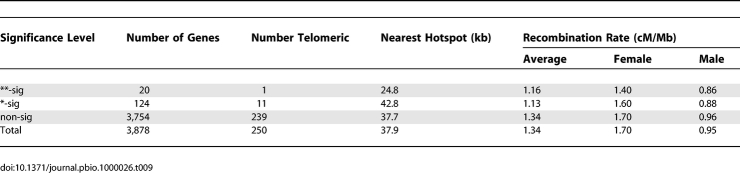
Recombination Rate in Genes with Evidence for Positive Selection Based on MK Tests

### Comparison of Fast-Evolving Genes Identified by Different Approaches

**Figure 5:**
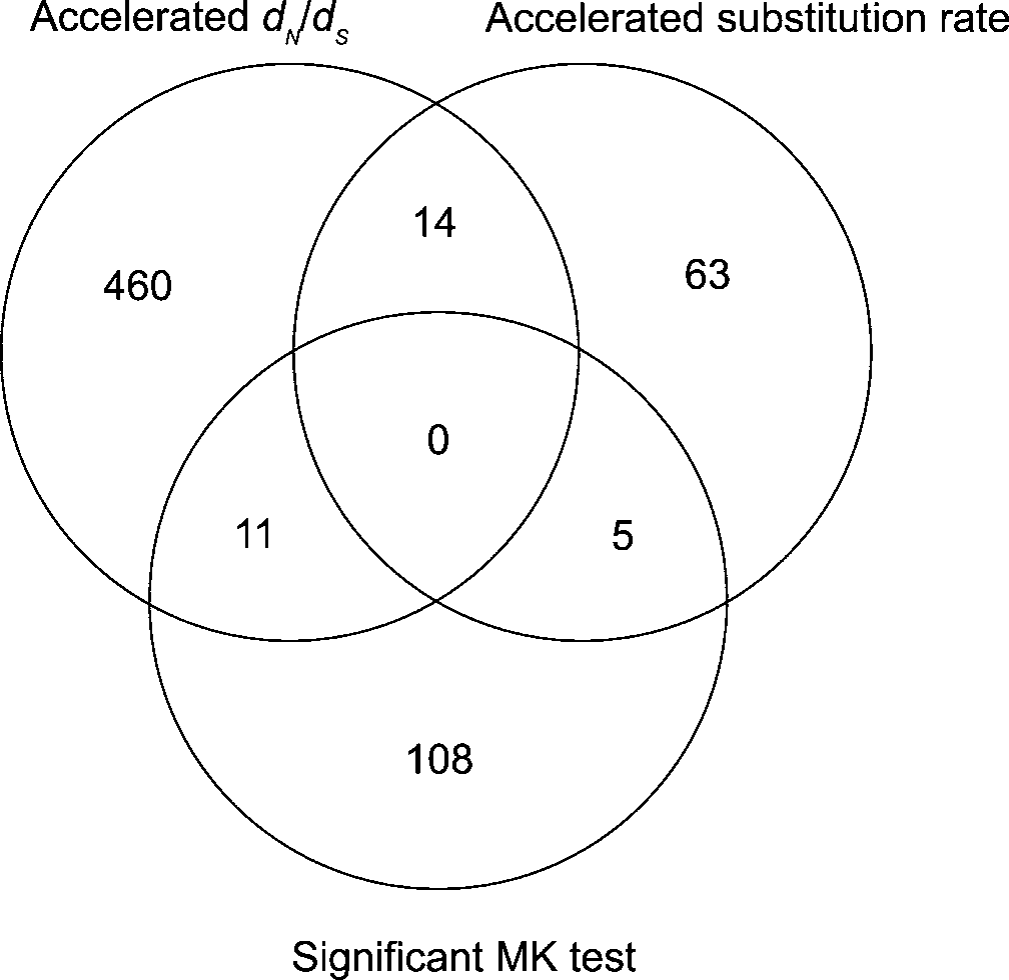
Venn Diagram Showing Overlap between Three Different Subsets of Fast-Evolving Genes Genes with evidence for accelerated *d_N_/d_S_* on the human lineage based on a LRT p<0.05 using a chi-squared test are shown in one circle. Genes containing exons with evidence for accelerated evolutionary rate in humans based on simulations with a FDR *p* < 0.05 are in the second circle. Genes with evidence for a significant McDonald-Kreitman test [39] with *p* < 0.05 are in the third circle.

Figure 5 indicates the overlap between the main sets of fast-evolving genes we have identified. Fourteen genes with accelerated *d*_N_ */d*_S_ on the human lineage at the *p* < 0.05 level also contain accelerated exons. This is significantly higher than the 3.9 genes expected purely by chance (binomial test *p* = 3.0 × 10^−5^), although it is not surprising that genes with evidence for acceleration in the relative rate of nonsynonymous substitutions also show evidence for acceleration in evolutionary rates overall. Eleven of the genes with accelerated *d*_N_ */d*_S_ also show evidence for an excess of amino acid substitutions using the MK test [39] at the *p* < 0.05 level, which is larger than the 5.9 expected, but not significant (*p* = 0.051). Five of the genes with significant MK tests also contain accelerated exons, which is larger than the 2.0 expected, but not significant (*p* = 0.054). The three different tests therefore have a tendency to identify some of the same genes, but in general they appear to target genes with different evolutionary histories.

### Enrichment for Gene Ontology Categories

Out of the 82 genes containing accelerated exons, the gene ontology (GO) category “myosin complex” is enriched (*p* = 0.0011) due to the presence of five myosin complex protein coding genes (*MYOM1*, *MYO18B*, *MYO3B*, *MYH10*, and *MYH3*). The genes containing the top 20 accelerated exons contain three olfactory receptors (*OR3A3*, *OR3A2*, and *OR4K17*), which generates a significant enrichment for “neurological system process” (*p* = 0.039). The GO category “multicellular organismal process” is strongly enriched in all of the genes containing accelerated exons (*p* = 5.09 × 10^−5^), as well as those containing the top 20 accelerated exons (*p* = 0.039).

We tested the genes with accelerated *d*_N_ */d*_S_ for enrichment of particular GO categories. There is no evidence for enrichment for any GO category at the *p* < 0.1 level for any of the accelerated *d*_N_ */d*_S_ significance levels. We also tested for enrichment of GO categories within genes with significant MK tests at the *p* < 0.05 level (190 genes). There was a significant enrichment for “calcium ion binding” among genes with evidence of recent positive selection (21 genes, *p* = 0.027).

### Minimal Effect of Ancestral Misinference

Another cause of apparent W→S substitutions could be misinference of ancestral bases. This is particularly problematic at CpG sites, where multiple CpG mutations on different lineages (always S→W) could give a false inference of the reverse (W→S) substitution due to homoplasy. In order to quantify this, we performed BLAST searches against three additional closely related primate genomes (gorilla, orangutan, and baboon) for the genes containing the top 20 accelerated exons, and the genes with strongest evidence (*p* < 0.001, ***) for accelerated *d*_N_ */d*_S_. All of the bases in both of these datasets were alignable to at least one of the three primates. We were able to align 95% of bases to at least two of the species and 84% of bases to all three species.

We compared the human-chimpanzee ancestral bases, inferred from the human-chimpanzee-macaque alignments by ML, to the orthologous bases in the additional primate genomes. We did not identify a single case where the ML inferred ancestral base of a human-specific substitution was incongruent with the orthologous base in the most closely related primate species. This indicates the effect of ancestral misinference is negligible in the human-accelerated sequences.

### Modeling the Effect of a W→S Fixation Bias on the *d*_N_/*d*_S_ Ratio

Our empirical results suggest that genes with elevated rates of nucleotide substitution have been affected by a fixation bias in favor of W→S mutations. Such a bias could be caused by BGC or directional selection on GC content. We also observe W→S biased substitution patterns in genes with (a) accelerated *d*_N_/*d*_S_ ratios on the human lineage, and (b) elevated numbers of nonsynonymous changes in substitution versus polymorphism data in MK tables. This suggests that a W→S fixation bias could potentially generate an increased rate of nonsynonymous compared with synonymous substitutions.

To investigate this issue, we used a theoretical model of the interaction between a W→S fixation bias and purifying selection in an ideal Wright-Fisher population with parameter values determined empirically from the exon dataset. We assume that a W→S fixation bias can be modeled using a selection coefficient, as demonstrated by Nagylaki [7]. We estimated the pattern of mutation in genes with different ancestral GC contents by analyzing substitutions in 4-fold degenerate (4d) sites. We used these to estimate the relative number of mutations expected in each mutational class, given the ancestral sequences. We then calculated the probability of fixation of each mutation, based on the selective coefficient and effective population size (*N*_e_; assumed to be 10,000). The selective coefficients were determined by combining a bias (*f*) that favors fixation of all W→S mutations and loss of all S→W mutations with a distribution of negative fitness effects on nonsynonymous mutations (*c*) derived by Eyre-Walker et el. [40]. We calculated the predicted substitution rate in each mutational class from the product of mutation and fixation probabilities.

**Figure 6:**
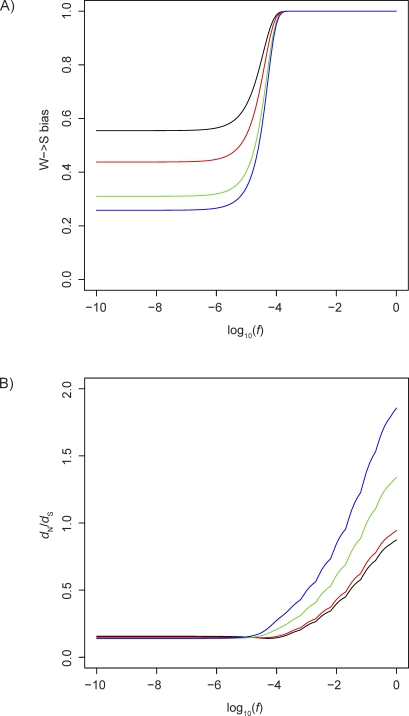
Predicted Effect of a Bias Towards Fixation of W→S Mutations, Considered as a Selective Coefficient (*f*), on Coding Sequences under Purifying Selection (A) Effect of *f* on the pattern of nucleotide substitutions (B) Effect of *f* on the *d_N_/d_S_* ratio The line colors represent ancestral GC content (0.3–0.4, black; 0.4–0.5, red; 0.5–0.6, green; 0.6–0.7, blue).

As expected, increasing the fixation bias (*f*) in favor of W→S mutations results in a W→S bias in the pattern of substitution (Figure 6A). This effect is most pronounced for GC-poor genes, whose substitution patterns have a higher degree of W→S mutational bias in the absence of a W→S fixation bias. A significant effect of *f* on the W→S bias can be observed once *f* > 1/4*N*_e_ (2.5 × 10^−5^), which is the approximate selection coefficient required for a new mutation under selection to have a higher probability of fixation than a neutral mutation. For values of *f* > 10^−4^, the W→S bias approaches 1.

Perhaps more surprisingly, at values of *f* > 10^−4^, W→S fixation bias is also predicted to increase the *d*_N_/*d*_S_ ratio (Figure 6B). Hence, when *f* is high it appears to override the effects of negative selection, leading to an increased proportion of nonsynonymous fixations. Interestingly, the effect of *f* is much more pronounced on GC-rich genes, and can potentially lead to *d*_N_/*d*_S_ > 1 (typically assumed to indicate positive selection). This phenomenon can be explained by an observation that W→S mutations in GC-rich genes have a greater probability of occurring in nonsynonymous sites. In GC-poor genes (ancestral GC = 0.3–0.4), we predict 47% of new W→S mutations to be nonsynonymous, whereas in GC-rich genes (ancestral GC = 0.6–0.7), we predict this proportion to be 66%. This difference is likely due to synonymous sites in GC-rich genes already being saturated with W→S substitutions. The effect of a W→S fixation bias is predicted to have a similar effect on genes under different levels of selective constraint (Figure S3).

## Discussion

We have identified a large number of exons with significantly accelerated evolutionary rates on the human lineage. The pattern and distribution of nucleotide substitutions in these exons suggests that a recombination-associated process, such as BGC [6] or strong localized selection in favor of increased GC content, is responsible for accelerated substitution rates in fast-evolving exons. The main support for this hypothesis is (a) an excess of W→S substitution in accelerated exons, (b) similar patterns of W→S bias in noncoding sequence flanking these exons, and (c) an enrichment of accelerated exons in regions of elevated male recombination and near the ends of chromosome arms. Accelerated exons also have an excess of replacement amino acid substitutions on the human lineage, which suggests that the process governing their evolution can compete with purifying natural selection.

### W→S Biased Evolution of Protein-Coding Sequences

Homologous recombination events between a pair of chromosomes that are heterozygous at a particular locus can lead to the formation of a heteroduplex DNA molecule during meiosis. The BGC hypothesis proposes that when the heteroduplex contains a weak/strong (AT/GC) mismatch, this is preferentially repaired to the strong allele [6]. This implies that weak/strong heterozygotes transmit more GC than AT alleles to the next generation, particularly at loci in regions of high recombination. Theoretical modeling has shown that this leads to biased fixation of W→S substitutions, with dynamics similar to natural selection [7], consistent with the patterns of evolution we observe in accelerated exons.

An alternative hypothesis is that directional selection for increased GC content has been operating on accelerated exons and their flanking regions. Clusters of highly expressed genes have been found to occur in regions of elevated GC content [41,42], and it is therefore possible that GC content has a direct effect on gene expression [43]. Hence, another explanation for our findings is that selection for increased gene expression has driven a local increase in GC content in the accelerated exons. mRNA structure and isochore GC content [30] are other possible sources of selective pressure. Without deeper understanding of the role of GC content in gene expression and chromosome evolution, it is difficult to hypothesize why selection on GC content would affect single exons and their flanking sequences rather than chromosomal domains or spliced transcripts.

Across the entire genome, clusters of human and chimpanzee nucleotide substitutions have a significant tendency to be W→S biased and to occur in regions of elevated recombination [20]. Such clusters are also observed in conserved noncoding elements with evidence for acceleration in humans (HARs) [1,5]. Here we have shown that protein-coding sequences are also subject to this unusual phenomenon. Exons with elevated substitution rates in humans exhibit a striking excess of W→S biased substitutions compared with all exons in the dataset. These substitutions also show a strong tendency to be clustered in single exons, rather than the entire gene. Similar W→S biased patterns can be observed in surrounding noncoding sequence, decaying sharply to background levels with increasing distance from the accelerated exon, so that W→S bias in 10 kb on each side of a accelerated exons approaches the genome average.

The observation that W→S biased substitution patterns extend into surrounding noncoding sequence strongly suggests that natural selection acting at the protein level could not be responsible for the biased fixation of GC alleles. However, it appears that clusters of W→S substitutions in accelerated exons are extremely localized to within a few kilobases around the exon. We also observe that when genes have evidence for clustering of base substitutions in a single exon, these substitutions exhibit a strong W→S bias. Furthermore, there is a strong discordance between levels of W→S bias in polymorphism compared with divergence, indicative of a bias towards fixation of W→S mutations. All of these observations are consistent with the action of BGC or localized selection in favor of increased GC content driving the evolution of the human-accelerated coding sequences we have identified.

It should be noted that not all accelerated exons have W→S biased substitution patterns. In particular, adenylate cyclase-associated protein 1 (CAP1) on human chromosome 1p34 contains a cluster of seven substitutions that are all S→W in exon 7. Six of these substitutions are nonsynonymous. None of the substitutions appear to be the result of CpG hypermutability. Furthermore, there is no evidence of a local increase in base substitution rate in the noncoding regions flanking exon 7; there are no substitutions on the human lineage within 100 bp of noncoding sequence on each side of this exon. It is possible that human-specific positive selection has contributed to acceleration in evolutionary rate in this exon. However, the biased pattern of base substitution suggests a bias in the pattern of mutation or fixation has also contributed, although the cause of this bias is unclear. Our method for analyzing accelerated evolutionary rates in single exons could potentially be a promising approach for identifying genes involved in human-specific adaptations.

### The Effect of Recombination Hotspots on Genome Evolution

Accelerated exons show a highly significant tendency to occur in regions of the human genome with elevated male recombination, consistent with the results of Dreszer et al. [20]. A correlation between the proportion of W→S substitutions and male recombination rate in humans is also observed in studies using human-chimpanzee-macaque comparisons across the genome [10], and in patterns of evolution in Alu repeats [9]. In addition, we find a significant enrichment of accelerated exons close to telomeres, similar to what has been observed for clusters of base substitutions in general and for HARs. Furthermore, like HARs [1,2], accelerated exons tend to be closer to recombination hotspots.

Most recombination in humans is restricted to short (<1 kb) hotspots where recombination rates are >10× the genomic average [28]. If BGC were able to generate nucleotide substitutions, we would expect them to be concentrated in these regions. Selection in favor of increased GC content would also be expected to be more efficient in these regions, due to a reduction in Hill-Robertson interference [33]. Recombination hotspots are believed to arise rapidly and become rapidly extinguished, potentially because of the “hotspot conversion paradox” [44]. Hotspot turnover is suggested by differences in the location of hotspots between human and chimpanzee [45,46], effects of certain allelic variants on recombination rate in known hotspots [47,48], and theoretical modeling [49]. In contrast, large-scale recombination rates are correlated between humans and chimpanzees [46]. Assuming that a strong W→S fixation bias is associated with recombination hotspots, then localized clusters of W→S substitutions would be expected to occur in bursts, mirroring the rapid turnover of recombination hotspots.

Due to their ephemeral nature, it is extremely difficult to measure the impact of hotspots in a particular genomic region since the human-chimpanzee ancestor. The two measures we used are large-scale recombination rates estimated from pedigree data [17] and a map of human recombination hotspots generated by analyzing patterns of linkage disequilibrium in the HapMap dataset. Estimates of the regional recombination rate and distance from the nearest hotspot of a particular genomic location are only rough indicators of the average density of recombination hotspots in that region since the human-chimpanzee split. Another problem is that we only have measures of when recombination events are resolved as crossovers and cannot directly measure the frequency or length of gene conversion events. Hence, although the clusters of substitutions we observe in accelerated exons are consistent with the action of an intense W→S fixation bias in recombination hotspots, we cannot reconstruct or implicate the locations of specific ancient hotspots.

We also observe W→S biased substitution patterns in the chimpanzee lineage for exons that are accelerated in humans, although the degree of bias is weaker. This suggests that high rates of recombination could also affect these exons in the chimpanzee lineage. Analysis of nucleotide substitutions across the entire human and chimpanzee genomes show similar patterns [10]. These findings are consistent with conservation of the average density of hotspots between human and chimpanzee, which could affect patterns of substitution in both lineages. It should be noted that we do not expect W→S fixation bias due to selection or BGC to be specific to the human lineage, and we would generally expect different exons to be accelerated on the chimpanzee lineage.

It is unclear why male recombination shows a stronger correlation with clusters of W→S biased substitutions (and with the pattern of W→S biased substitution across the genome) than does female recombination. This difference is not predicted by a model of selection on GC content modulated by Hill-Robertson effects on the efficacy of selection. It is possible that there is a stronger correspondence between the rate of crossovers and gene conversion in males than in females, which would cause the strength of BGC to correlate more strongly with male recombination.

The site frequency spectrum should be altered in regions where a W→S fixation bias is occurring, due to an increase in frequency of GC alleles. Several studies have demonstrated that GC alleles segregate at elevated frequencies across the genome in human populations, and that this effect is stronger near recombination hotspots [21,26,50]. However, it has been suggested that these findings may have been influenced by a systematic error in inferring the ancestral state of SNPs [27]. An alternative theory for how recombination could generate W→S substitutions is by a direct mutagenic effect [23,51]. However, neither ancestral misinference nor a mutagenic effect of recombination can explain why the excess of GC alleles at elevated frequency increases in the vicinity of recombination hotspots [10]. In particular, the allele frequency distribution at a recently arisen recombination hotspot should be skewed towards low frequency GC alleles if recombination generated W→S mutations, which is the opposite of what is observed. A recent model of the effect of BGC on the pattern of base substitution [10] is a good fit to observed patterns across the human genome, suggesting that BGC—rather than a direct effect of recombination—can account for the patterns of molecular evolution observed in the human-accelerated coding regions we have identified. It is also possible that a reduction in Hill-Robertson interference in regions of high recombination could result in clusters of W→S biased substitutions due to selection. However, it is currently unclear whether this process would result in the observed variation in W→S bias and substitution rate over short physical distances.

### W→S Fixation Bias Drives Amino Acid Replacement Substitutions

An important finding of this study is that biased fixation of W→S mutations can drive replacement amino acid substitutions. In addition to W→S biased substitution patterns, accelerated exons exhibit an excess of nonsynonymous to synonymous changes compared with the genomic average. This is consistent with previous suggestions that BGC may compete with purifying selection, resulting in the fixation of deleterious mutations [5]. This process may have occurred in noncoding HARs, which are generally extremely highly conserved between mammalian species other than humans, and probably play important functional roles (e.g., in regulation of gene expression). An increase in mutation rate is not expected to increase the proportion of nonsynonymous to synonymous changes, as it would be expected to remove the same proportion of nonsynonymous changes in regions of high or low mutation rates. Natural selection on amino acid sequence is also not expected to generate the W→S biased substitution patterns we observe in accelerated exons, which extend into noncoding flanking sequence.

Using theoretical modeling, we have shown that W→S fixation bias is predicted to increase the *d*_N_/*d*_S_ ratio under realistic assumptions regarding the strength of bias and distribution of negative fitness effects on nonsynonymous mutations. This effect is observable with a fixation bias corresponding to a selective coefficient >10^−4^ (assuming *N*_e_ = 10,000). An important simplifying assumption made by our model is that the W→S fixation bias can be modeled in an identical way to positive selection [7]. In reality there is likely to be a complex interaction between the two processes. In particular, the effects of selection may extend to distant linked sites, whereas the effects of BGC are likely to be confined to short conversion tracts. Further work is necessary to fully understand this interaction. Our model uses the method of Li [52] to predict the *d*_N_/*d*_S_ ratio, whereas we used a codon-based ML method [53] to estimate this ratio from our alignments. Although the two methods may give slightly different estimates of *d*_N_ and *d*_S_ under certain scenarios, we do not expect this discrepancy to influence our prediction that a W→S fixation bias increases the *d*_N_/*d*_S_ ratio. One assumption of codon models of substitution is that codon frequencies are at equilibrium, which is unlikely to be true at loci where a fixation bias is operating. However, the choice of model is not likely to lead to misinference of ancestral bases at short genetic distances, such as between human and chimpanzee.

The strength of BGC depends on the rate of formation of heteroduplex DNA, the size of the hetoroduplex tracts, and the strength of the repair bias, all of which are difficult to estimate empirically. By comparing a large number of studies in yeast, Birdsell [15] has estimated a that GC/AT mismatches are repaired to GC with a bias of about 1.5. Recombination rates of >50 cM/Mb are predicted to be common in localized (1–2 kb) hotspots in the human genome [28], and even higher rates have been observed in individual hotspots [54]. We cannot predict how often a particular site in a recombination hotspot will be involved in biased repair from these figures. However, it does not seem unrealistic that fixation biases >10^−4^ could occur at particular sites due to BGC if they are regularly included in recombination events in hotspots.

### Function of Genes Containing Accelerated Exons

We observed enrichment of certain GO categories in genes containing accelerated exons. Olfactory receptor proteins were overrepresented in the top 20 accelerated exons, and the genes containing the 83 accelerated exons were enriched for myosin complex genes. It is notable that these genes belong to superfamilies with many paralogs. In addition to allelic gene conversion events between homologues, gene conversion also occurs between duplicated paralogous genes. Previous studies have suggested that gene conversion between paralogs generates W→S biased patterns of base substitution in gene families [11,12]. Hence it is possible that extremely high levels of BGC between paralogs has contributed to the accelerated evolution and biased patterns of substitution we observe. Gene conversion is likely to occur more frequently between physically linked gene duplicates [11]. It is interesting to note that two of the fast-evolving olfactory receptors, *OL3A3* and *OL3A2*, lie within 200 kb of each other on the last band of chromosome 17p, close to a number of other olfactory receptors. Although none of the myosin complex genes containing accelerated exons occur on the same chromosome, *MYH3* lies within a cluster of myosin family genes spanning 300 kb on chromosome 17p13.

Olfactory receptors are part of the largest supergene family in mammals. Several of these genes are believed to be under positive selection in humans, although disproportionately large numbers have become pseudogenes in humans compared with chimpanzee [55]. It is possible that excess fixations caused by BGC in human olfactory receptors are tolerated because purifying selection is relaxed in these genes in humans, and in some cases this has resulted in pseudogenization. However, it is also possible that previous reports of an enrichment of olfactory receptors amongst genes with accelerated *d*_N_ */d*_S_ on the human lineage [56] could be influenced by BGC. Because our dataset is constructed from 1:1:1 orthologues, we cannot observe the effects of gene conversion between recent duplicates. However, BGC between closely related paralogs could potentially have a major influence on their evolution.

### Evolution of *KIF26B*

One gene, kinesin family member 26B (*KIF26B*), shows a particularly striking pattern of evolution on the human lineage. The most accelerated exon of this gene is exon 1, which is 718 bp long and has 17 substitutions on the human lineage. All of these substitutions are W→S and nonsynonymous. The remaining three exons lie > 9 kb away from the first and have just two substitutions (one W→S and one S→W) within 500 bp. This gene is also the only one with a highly significantly accelerated *d*_N_ */d*_S_ (***) that also contains one of the top 20 accelerated exons.

The molecular evolution of *KIF26B* in primates strongly parallels the *Fxy* gene in rodents. The 3′ portion of *Fxy* has been translocated into the highly recombining pseudoautosomal region (PAR) in M. musculus, whereas in the closely related M. spretus the entire gene is nonrecombining. This translocation coincides with a massive increase in the substitution rate in the 3' end of *Fxy*; M. musculus has 28 nonsynonymous substitutions, all of which are W→S, compared with only one nonsynonymous substitution in M. spretus [14]. The human substitutions at *KIF26B* are also almost exclusively nonsynonymous (18 out of 19) and the ML inferred *d*_N_ */d*_S_ ratio for the human branch is 1.75. This extreme substitution pattern may indicate the involvement of both positive selection and BGC. However, *KIF26B* has an extremely high GC content (0.72), and our theoretical modelling suggests that a W→S fixation bias can potentially result in *d*_N_ */d*_S_ > 1 in such GC-rich genes in the absence of positive selection. This is because synonymous sites are more saturated with W→S substitutions in GC-rich genes so that W→S mutations have a greater probability of occurring in nonsynonymous sites, which could also explain why the W→S bias is greater in nonsynonymous sites in accelerated exons. It is possible that positive selection can promote compensatory amino acid replacement substitutions after deleterious mutations become fixed due to BGC, but these substitutions would not all be expected to be W→S, as observed at the *KIF26B* locus.

### W→S Fixation Bias Can Influence Tests of Positive Selection

We identified genes with evidence for increased rates of *d*_N_ */d*_S_ on the human lineage using an LRT. We find that the most diverged exons in these genes have significantly W→S biased substitution patterns. This pattern is not seen in the most diverged exons of genes overall, indicating that W→S fixation bias may affect the evolution of the genes with the strongest evidence for accelerated rates of amino acid substitutions in humans. The strongest signal for W→S bias occurs in the exons with the largest proportion of amino acid replacement substitutions. These observations suggest that a W→S fixation bias could contribute to elevated levels of *d*_N_ */d*_S_ and possibly lead to false inference of positive selection at the protein level.

Genes with accelerated *d*_N_ */d*_S_ differ from accelerated exons in several ways. First, we do not observe significantly W→S biased substitution patterns in the noncoding regions associated with genes with accelerated *d*_N_ */d*_S_. Nonetheless, the significantly elevated GC content of these regions suggests that they may have experienced W→S biased substitution patterns previously. Second, compared to accelerated exons, genes with accelerated *d*_N_ */d*_S_ show a weaker association between substitution rates and local recombination rates. These genes are significantly enriched close to human recombination hotspots, but they are not enriched in regions of elevated recombination (measured from the DECODE recombination map [17]) or in distal chromosome regions. These findings suggest that while the signature of recombination-associated W→S fixation bias is observable within genes with elevated *d*_N_ */d*_S_ in humans, they have been affected to a lesser extent than the accelerated exons. Alternatively, W→S biased substitution patterns in genes with accelerated *d*_N_ */d*_S_ may result from different evolutionary processes. For example, one factor that could contribute to the relationship between recombination hotspots and *d*_N_ */d*_S_ is the higher efficiency of natural selection in regions of high recombination, due to a reduction in the strength of Hill-Robertson interference [33].

We also observed an increase in W→S bias in genes with an excess of amino acid replacement substitutions relative to human polymorphism identified using the modified MK test [35] of Bustamante et al. [39]. The largest W→S bias occurs in genes with the most significant values of the test. W→S bias is particularly marked in the most diverged exon of each gene. These genes are also significantly enriched close to human recombination hotspots, although their average recombination rates are not significantly different from the genomic average. This pattern is consistent with a W→S fixation bias driving additional weakly deleterious amino acid substitutions, mainly restricted to single exons. Our observations are consistent with the past existence of transient recombination hotspots in these regions, which are, in general, no longer actively driving fixation of GC alleles in the human population. However, as we do not observe a strong correlation between present day recombination rates and significant MK test results, it is possible that the W→S fixation bias is unrelated to recombination in these genes.

Overlap between genes identified by the two tests of positive selection is not significantly higher than expected by chance, which is indicative that they identify genes with different evolutionary histories. The *d*_N_ */d*_S_ LRT identifies genes with increased *d*_N_ */d*_S_ ratios on the human lineage. These genes would not necessarily exhibit a significant excess of amino acid substitutions relative to polymorphism, unless they were under the influence of strong positive selection, or experienced recent shifts in their mode of evolution. In contrast, the MK test compares patterns of divergence and polymorphism, but does not detect whether the rate of protein evolution has changed on the human lineage. The accelerated exons do not all show a strong excess of nonsynonymous changes, which explains the limited overlap between them and genes identified by tests of selection. It is notable that all three tests identify coding sequences with W→S biased patterns of substitution.

The effect of W→S fixation bias at a particular locus is likely to depend on a variety of factors, including the time scale and intensity of a W→S drive and the locus-specific interaction with natural selection. All of these factors are predicted to vary between loci, likely due to stochastic variation in the strength and location of recombination hotspots over time. It is therefore not surprising that signals of a W→S fixation bias can be found using a variety of tests for increased evolutionary rates. Importantly, our results suggest that a W→S fixation bias, rather than positive selection on protein function, could be responsible for generating significant tests for selection in some genes, which cause us to urge care in the interpretation of these tests.

###  Conclusion

We have presented evidence that protein-coding sequences with accelerated rates of evolution in humans have significantly biased patterns of nucleotide substitutions. These results are consistent with a strong effect of W→S fixation bias on the evolution of the most rapidly evolving coding exons in our genome. This process may have led to the increased fixation of replacement amino acid changes on the human lineage, and may bias tests of positive selection.

## Materials and Methods

### Data.

We analyzed a dataset of 10,376 alignments of 1:1:1 human-chimpanzee-macaque orthologous genes presented in the rhesus macaque genome paper [36] and available from http://compgen.bscb.cornell.edu/orthologs/. The alignments consisted of a filtered dataset of orthologous genes derived from known human protein coding genes identified from the RefSeq [57], Vega [58], and UCSC known gene [59] annotations. Genes with poor syntenic relationships, incomplete alignments, frame-shift indels, changes in exon-intron structure, and evidence for recent duplications had all been excluded from the dataset [36]. Annotation files for all of the genes were downloaded from Biomart (http://www.ensembl.org/biomart/martview/) and UCSC (http://genome.ucsc.edu/). These were used to identify the exon boundaries in all of the alignments and their location in the hg18 human genome sequence assembly. A small number of genes (<1%) were excluded due to poorly matching gene annotation data. We finally excluded alignments with ten or more bases in runs of mismatches of three or more between any of the sequences, resulting in a dataset of 10,238 genes.

Human recombination rates, based on the DECODE map [17] were obtained from the UCSC table browser (http://genome.ucsc.edu/cgi-bin/hgTables). Positions of human recombination hotspots as inferred from coalescent analysis of large-scale SNP genotyping data were downloaded from the HapMap website (http://www.hapmap.org/downloads/). All analyses were based upon the hg18 human genome assembly, aligned to the chimpanzee (panTro2) and the macaque genome (rheMac2). The liftover tool, available from the UCSC website, was used to convert human annotation to the hg18 assembly where needed (http://hgdownload.cse.ucsc.edu/downloads.html).

We also constructed alignments of the noncoding sequence flanking each exon in the coding dataset. To do this, we first obtained pairwise chained and netted blastz alignments of the hg18 versus panTro2 assemblies and the hg18 versus rheMac2 assemblies from the UCSC website (http://hgdownload.cse.ucsc.edu/downloads.html). These were converted into a human-chimpanzee-macaque alignment for each human chromosome using tools from the multiz package [60]. Finally, we masked all exons and extracted flanking sequence on both sides of each exon using the gene annotation files.

### Analysis of nucleotide substitutions.

We used the phast package [61] to analyze the rate of nucleotide substitution individually for each exon sequence alignment, implementing the general time-reversible (REV) model. We compared a model with relative branch lengths (i.e., relative substitution rates) equal to those from a genome-wide model to a model where the human branch is longer (i.e., has an accelerated substitution rate). We used an LRT to identify exons with statistically significant substitution rate acceleration on the human branch [1].

We used the codeml program of PAML [62] with F3x4 codon frequencies and the Goldman and Yang [53] model of codon substitution to infer the pattern of synonymous and nonsynonymous nucleotide substitutions at each gene on the human and chimpanzee branches of the tree under two models usingML. We first used the one-ratio model, where the *d*_N_ */d*_S_ ratio was fixed along all lineages of the tree. We compared this with the two-ratio model, were *d*_N_ */d*_S_ was allowed to vary along the human lineage. Sequences with accelerated *d*_N_ */d*_S_ on the human branch were identified with an LRT. We repeated this analysis on the whole-gene alignments and on individual exon alignments. For the single exon analysis, codons that overlapped between two consecutive exons were removed from the alignments.

We compared the ML reconstructed human-chimpanzee ancestral sequence from the two-ratio model with the human sequence to determine the pattern of substitutions along the human lineage. Substitutions were divided into four different classes: strong-to-strong (S→S), strong-to-weak (S→W), weak-to-strong (W→S), and weak-to-weak (W→W). “Weak” designates A or T base pairs, which are bound by only two hydrogen bonds. “Strong” designates C or G base pairs, which are bound by three hydrogen bonds. We defined the W→S bias of a genomic region as follows: W→S bias = *n*_W→S_ / (*n*_W→S_ + *n*_S→W_), where *n*_W→S_ and *n*_S→W_ are the number of W→S and S→W substitutions, respectively. Substitutions were also classified as nonsynonymous or synonymous. Multiple substitutions in the same codon were taken into account using the same criteria as single substitutions. These were inferred by ML to account for only 0.9% of substitutions. Substitutions in noncoding alignments surrounding each exon, and the GC content of the ancestral sequences, were inferred using parsimony. Human-specific substitutions were identified and assigned to the four different categories above by determining the ancestral state of each site using the macaque sequence.

We analyzed whether there was a tendency for nucleotide substitutions to cluster using a statistic that captures the relative substitution rate in the most diverged exon of a gene compared to the overall substitution rate in the gene:


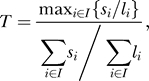


where 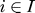 indexes the exon, *s_i_* is the number of single base substitutions in exon *i,* and *l_i_* is the length of exon *i* (in bases)*.* Genes with large values of *T* have an exon that has a higher substitution rate than expected given the overall rate of substitutions across exons in the gene. For each gene, we conducted a simulation to assess the statistical significance of the observed value of *T*. Fixing the exon boundaries at the observed positions, we uniformly placed the observed number of substitutions 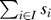 at random sites across the gene and calculated the value of *T*. Repeating this substitution assignment 1,000 times provides a null distribution for the statistic *T*, under the assumption that the substitution process is uniform. An empirical *p*-value can be calculated as the proportion of the 1,000 null *T* values that exceed the observed *T* value.

### Identifying accelerated evolution in humans.

We estimated the total number of substitutions of each type for every gene, dividing substitutions into individual exons. We also calculated the GC content of each exon, the distance to the nearest recombination hotspot, the recombination rate, and whether each gene is in the last chromosome band. We ranked each exon according to its degree of acceleration in evolutionary rate on the human lineage, taking all substitutions into account, using the LRT statistic with the REV model of nucleotide substitution. Significance was estimated by simulating 10,000 datasets from the null model and calculating the LRT statistic for each exon. The *p*-value for each exon was estimated as the number of simulated LRTs that exceed the observed value. These p-values were adjusted for multiple testing using the FDR controlling method of Benjamini & Hochberg [37].

We also classified genes according to evidence of a significantly different *d*_N_ */d*_S_ along the human lineage. We divided genes into three levels of significance based on comparing the LRT statistic to the chi-square distribution: *p* < 0.001 (***), *p* < 0.01 (**), and *p* < 0.05 (*). We ranked exons according to their level of “relative divergence,” using the statistic *T* defined above. Significant differences in the patterns of nucleotide substitution in the most accelerated exons and genes in all categories were identified using FET and by bootstrapping each exon with 10,000 replicates. The FET assumes that each substitution is an independent data point, whereas the bootstrap test considers each exon independently thereby accounting for correlation between substitutions within an exon.

### Comparison with human polymorphism.

We identified human SNPs within our alignments using data from the HapMap project. We obtained the position and alleles of >15 million SNPs on the hg18 human genome build using the HapMart tool available at http://hapmart.hapmap.org/BioMart/martview. We then determined the ancestral allele of each SNP that overlapped one of our alignments by comparison with the chimpanzee base at that position. To minimize errors due to ancestral misinference, only biallelic SNPs where one allele matched both the chimpanzee and macaque sequence were included in the analysis.

We identified genes in our dataset with evidence for an excess of amino acid replacement substitutions relative to human polymorphism based on the genome scan of Bustamante et al. [39]. This analysis estimated the selection coefficient from MK contingency tables [35] of polymorphism and divergence at synonymous and nonsynonymous sites. The posterior probability of the selection coefficient was used to estimate the probability that a gene is under positive selection (has an excess of amino acid replacement substitutions). Only genes that appeared in our dataset and in the dataset of Bustamante et al. [39] were included in the analysis. We compared patterns of nucleotide substitutions between genes with evidence for positive selection with the entire dataset using FET and bootstrapping each gene with 10,000 replicates. The FET assumes that each substitution is an independent data point, whereas the bootstrap test considers each gene independently.

### Enrichment for GO categories.

We tested whether genes containing exons with significantly accelerated rates of base substitution, genes with significantly accelerated *d*_N_ */d*_S_ ratios, and genes with significant MK tests were enriched for particular GO categories. We performed these analyses using GOstat [63], available at the website http://gostat.wehi.edu.au/.

### Homology searches.

To detect potential incidences of ancestral misidentification in our dataset, we performed BLAST searches of the most accelerated genes and exons in our alignments against the NCBI trace archives from three other primate sequences: gorilla (Gorilla gorilla), orangutan (*Pongo pygmaeus abelii*), and baboon (Papio hamadryas). These sequences were aligned to the existing alignments and used to identify human-chimpanzee mismatches where the ML inferred ancestral base was incongruent with the orthologous base in the additional species. In cases where bases were not in concordance between the three additional species, the base from the species most closely related to human and chimpanzee was compared with the inferred ancestral base.

### Modeling the effect of a W→S fixation bias on coding sequence evolution.

Nagylaki [7] demonstrated that BGC can be modeled using a selection coefficient. We therefore assume that BGC and a W→S fixation bias due to selection can be modeled in the same way. We modeled the effect of a W→S fixation bias by applying the inferred neutral mutation rate, a realistic distribution of negative fitness effects on nonsynonymous sites, and a range of values of a selection coefficient that favors fixation of W→S mutations and loss of S→W mutations, to the inferred ancestral sequences. We first estimated the pattern of neutral mutation on the human lineage using the inferred pattern of substitution at fourfold degenerate sites from our codeml analysis with the two-ratio model (see above). The mutation pattern was estimated separately within four categories of ancestral GC content (0.3–0.4, 0.4–0.5, 0.5–0.6, 0.6–0.7). The relative probability of every possible mutation at each base in each GC content category was calculated as follows for each of the 12 possible single base mutations. The A→C mutation is shown as an example:


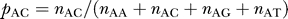


where *n*_X_ is the number of sites in category X and X is a mutational type (e.g., *n*_AC_ is the number of sites with A→C mutations).

We next concatenated the ancestral sequences of all genes in each GC content category. For each concatenated sequence, we calculated the expected relative rate of synonymous and nonsynonymous mutations in each of the following mutational classes (W→S, S→W, W→W, S→S) by adapting the method of Li [52] as follows. First, the number of nondegenerate (*L*_0_), 2-fold degenerate (*L*_2_), and 4-fold degenerate sites (*L*_4_) in the ancestral sequence were counted. A site is nondegenerate if all possible mutations at that site are nonsynonymous, 2-fold degenerate if one of the possible mutations is synonymous, and 4-fold degenerate if all possible changes are synonymous. The one possible case of a 3-fold degenerate site is treated as 2-fold degenerate. Each possible mutation at every site in the ancestral sequence was then classified as a transition or a transversion. The relative numbers of expected transitional (*S_i_*) and transversional (*V_i_*) mutations (*i* = 0, 2, 4) at each type of site in the entire sequence were then calculated separately for W→S, S→W, W→W, and S→S mutations as the sum of the relative probability of each possible transition and transversion at each site. Finally, we calculated the expected relative rate of synonymous and nonsynonymous mutations separately for each of the four mutational classes (*u*_WS(syn)_, *u*_SW(syn)_, *u*_WW(syn)_, *u*_SS(syn)_, *u*_WS(nonsyn)_, *u*_SW(nonsyn)_, *u*_WW(nonsyn)_, *u*_SS(nonsyn)_), using the values of *S_i_*, *V_i_*, and *L_i_* to estimate *d*_N_ and *d*_S_ according to Li [52]:

We simulated the selective coefficient (*s*) for each of the eight classes of mutation based on the combined effects of a W→S fixation bias (*f*) and selective constraint (*c*). The values of *s* for synonymous changes are: *s*_WS(syn)_ = *f*, *s*_SW(syn)_ = –*f*, *s*_WW(syn)_ = 0, and *s*_SS(syn)_ = 0. The W→S fixation bias alters the probability of fixation of W→S and S→W mutations, but W→W and S→S mutations are assumed to evolve completely neutrally. The values of *s* for nonsynonymous changes are: *s*_WS(nonsyn)_ = *f – c*, *s*_SW(nonsyn)_ = – *f – c*, *s*_WW(nonsyn)_ = –*c* and *s*_SS(nonsyn)_ = –*c*. Selective constraint on a nonsynonymous mutations depends on a distribution of negative fitness effects, which is commonly modeled using a gamma distribution. We therefore sampled *c* from a descretized gamma distribution with shape parameter 0.23 and mean 0.0425, assuming an effective population size (*N*_e_) of 10,000. This distribution was inferred by Eyre-Walker et al. [40] to be a good fit to the distribution of fitness effects of SNPs segregating in the human population, and is in good concordance with other studies (e.g., [64]). We considered a range of values of *f* between 10^−10^ and 1.

We calculated the probability of fixation separately for mutations in each of the 8 classes using the following equation derived by Kimura [65]:


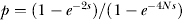


where *N* is the population size of an ideal Wright-Fisher population, which we assume to be 10,000. The predicted relative substitution rates (*K*) at each mutational class were then calculated by multiplying their probability of fixation, *P*, by their relative mutation rates, *u*. We calculated K for each value of *f* (between 10^−10^ and 1), separately for each ancestral GC content category. We used these rates to calculate *d*_S_, *d*_N_, and W→S bias by summing across the different mutational categories.

## Supporting Information

Figure S1 Genes Containing the Most Relatively Diverged Exons Exon boundaries are marked with black lines. S→W substitutions on the human lineage are marked with blue lines, W→S substitutions on the human lineage are marked with red lines and all other substitutions on the human lineage are marked with grey lines. Relative divergence is calculated as described in the methods.
(268 KB EPS)

Figure S2 Top 20 Genes with Strongest Evidence for Accelerated *d*_N_ */d*_S_ Based on LRT Exon boundaries are marked with black lines. S→W substitutions on the human lineage are marked with blue lines, W→S substitutions on the human lineage are marked with red lines and all other substitutions on the human lineage are marked with grey lines.
(262 KB EPS)

Figure S3 The Predicted Effect of Different Levels of Constraint on the Relationship between W→S Fixation Bias and *d*_N_ */d*_S_
A selective coefficient (*f*) is used to represent the W→S fixation bias. The level of constraint is fixed at the following values: (a) 2 × 10^−5^, (b) 4 × 10^−5^, (c) 8 × 10^−5^, (d) 1.6 × 10^−4^. The line colors represent ancestral GC content (0.3–0.4, black; 0.4–0.5, red; 0.5–0.6, green; 0.6–0.7, blue).
(294 KB EPS)

Table S1 LRT Statistics for Human Acceleration and Pattern of Substitution on the Human Lineage for All Exons (9.13 MB CSV)

Table S2 LRT for Accelerated *d*_N_ */d*_S_ on Human Branch Compared with Constant *d*_N_ */d*_S_ across the Tree for All Genes (327 KB CSV)

## Abbreviations

BGC: biased gene conversion
FET: Fisher's exact test
GO: gene ontology
HAR: human-accelerated region
LRT: likelihood ratio test
MK test: McDonald-Kreitman test
ML: maximum likelihood
PAR: pseudoautosomal region
W→S: weak-to-strong
